# The active component of ginseng, ginsenoside Rb1, improves erythropoiesis in models of Diamond–Blackfan anemia by targeting Nemo-like kinase

**DOI:** 10.1016/j.jbc.2021.100988

**Published:** 2021-07-21

**Authors:** Mark C. Wilkes, Kevin Jung, Britney E. Lee, Mallika Saxena, Ryan S. Sathianathen, Jacqueline D. Mercado, Cristina Perez, Johan Flygare, Anupama Narla, Bertil Glader, Kathleen M. Sakamoto

**Affiliations:** 1 Division of Hematology/Oncology, Department of Pediatrics, Stanford University, Stanford, California, USA; 2 Department of Molecular Medicine and Gene Therapy, Lund Stem Cell Center, Lund University, Lund, Sweden

**Keywords:** erythropoiesis, cell differentiation, cell signaling, ribosome, miRNA, Diamond–Blackfan anemia, ribosomopathy, CB, cord blood, DBA, Diamond–Blackfan anemia, HSPC, hematopoietic stem and progenitor cell, MAPK, mitogen-activated protein kinase, NLK, Nemo-like kinase, qRT, quantitative RT, RFP, red fluorescent protein

## Abstract

Nemo-like kinase (NLK) is a member of the mitogen-activated protein kinase family of kinases and shares a highly conserved kinase domain with other mitogen-activated protein kinase family members. The activation of NLK contributes to the pathogenesis of Diamond–Blackfan anemia (DBA), reducing c-myb expression and mechanistic target of rapamycin activity, and is therefore a potential therapeutic target. Unlike other anemias, the hematopoietic effects of DBA are largely restricted to the erythroid lineage. Mutations in ribosomal genes induce ribosomal insufficiency and reduced protein translation, dramatically impacting early erythropoiesis in the bone marrow of patients with DBA. We sought to identify compounds that suppress NLK and increases erythropoiesis in ribosomal insufficiency. We report that the active component of ginseng, ginsenoside Rb1, suppresses NLK expression and improves erythropoiesis in *in vitro* models of DBA. Ginsenoside Rb1–mediated suppression of NLK occurs through the upregulation of miR-208, which binds to the 3′-UTR of NLK mRNA and targets it for degradation. We also compare ginsenoside Rb1–mediated upregulation of miR-208 with metformin-mediated upregulation of miR-26. We conclude that targeting NLK expression through miRNA binding of the unique 3′-UTR is a viable alternative to the challenges of developing small-molecule inhibitors to target the highly conserved kinase domain of this specific kinase.

Nemo-like kinase (NLK) is an atypical member of the mitogen-activated protein kinase (MAPK) family of kinases and is 41.7% identical to extracellular signal–regulated kinase 2 and 38.4% with Cdc2 (1). The kinase domain is particularly conserved, especially with extracellular signal–regulated kinase 5, and many substrates of NLK are also phosphorylated by other family members, albeit with different kinetics (2). Consequently, there are currently no small-molecule inhibitors with high specificity for NLK. NLK is upregulated and correlated with poor prognosis in non–small-cell lung carcinoma and small-cell lung carcinoma, colorectal cancer, hepatocellular carcinoma, laryngeal cancer, osteosarcoma, and neuroblastoma (3, 4, 5, 6, 7, 8, 9, 10).

Diamond–Blackfan anemia (DBA) is a rare genetic disease. Over 80% of patients express a ribosomal gene mutation, with 25% carrying a mutation in RPS19 (11). Patients usually present in the first year of life with macrocytic anemia, requiring lifelong blood transfusions and steroid treatment (12). The hematologic symptoms can be cured by hematopoietic stem cell transplantation, but currently, only 16% of patients are transplanted because of the significant risks of life-threatening complications (11). NLK is activated in response to ribosomal insufficiency in DBA (2), and the suppression of NLK expression or activity (2, 13) significantly improves erythroid expansion. Therefore, the development of treatments that inhibit NLK promises significant clinical benefits for patients with DBA.

Despite there being no specific small-molecule inhibitor of NLK activity, the commonly prescribed diabetes drug, metformin, has demonstrated efficacy in downregulating NLK expression (9, 13) and improving erythropoiesis in human, mouse, and animal models of DBA (13). We previously determined that metformin-mediated suppression of NLK in DBA models was due to the upregulation of miR-26a (13). Because of the challenges of specifically targeting the kinase domain of active NLK, utilizing compounds that upregulate specific miRNAs that could specifically suppress NLK expression offers an alternative to small-molecule kinase inhibitors.

The expression of most mRNAs is modulated by the binding of miRNAs to the 3′UTR (14, 15, 16, 17). For many of these transcripts, the degree of modulation is moderate and likely represents a mechanism for tissue-specific fine tuning of expression. In contrast, NLK mRNA is particularly susceptible to significant miRNA-mediated downregulation, including downregulation by miR-199, miR101, miR-221, miR-26a, and miR-181 (18, 19, 20, 21, 22). As the sequence of the 3′UTR of NLK differs significantly from other MAPK family members, targeting NLK through this mechanism is unlikely to have off-target impacts on other MAPK pathways. As the miRNA upregulated in response to specific stimuli is often tissue specific (14, 16, 23), this could also reduce off-target effects compared with the systemic delivery of a small molecule.

Over 80% of patients with DBA carry germline ribosomal mutations that are expressed in all cell types, including other hematopoietic lineages (24). However, one reason the anemic phenotype is so pronounced is because NLK expression is dramatically higher in erythroid, compared with nonerythroid hematopoietic lineages. NLK is expressed in hematopoietic stem cells and early progenitors but is downregulated in nonerythroid lineages because of the upregulation of miR-181 (2). Indeed, the balance of miR-181 expression is critical in fate decisions between erythroid and megakaryocyte differentiation (25), but the evolutionary selective pressure to maintain the miR-181–mediated downregulation of NLK is unclear. Although NLK is neither activated nor required for normal erythropoiesis (2), the miR-181 binding sequence within the 3′UTR is evolutionarily conserved in humans, mice, and zebrafish, whereas significant divergence occurs in other regions. Whether NLK expression in erythropoiesis is selected for or not, upstream cues in DBA stimulate NLK activation, and because it is only expressed in erythroid cells, these are the cells that become burdened with the effects of NLK activation (2). While NLK expression is highly responsive to miR-181 expression, manipulating miR-181 levels would in addition deregulate multiple aspects of hematopoiesis.

Here, we report that ginsenoside Rb1 (the active component of ginseng) upregulates miR-208 in differentiating hematopoietic stem and progenitor cell (HSPCs). This upregulation targets NLK mRNA for degradation and reduces the amount of NLK protein available to be activated in ribosome insufficiency, thereby improving erythroid expansion in these cells. As this represents the second compound that utilizes this mechanism of action, we propose that targeting NLK expression with miRNAs is a viable alternative to small-molecule kinase inhibitors that will likely be nonspecific with significant off-target effects.

## Results

### Ginsenoside Rb1 improves erythroid expansion without impacting myeloid expansion in *in vitro* model of DBA

**Figure 1:**
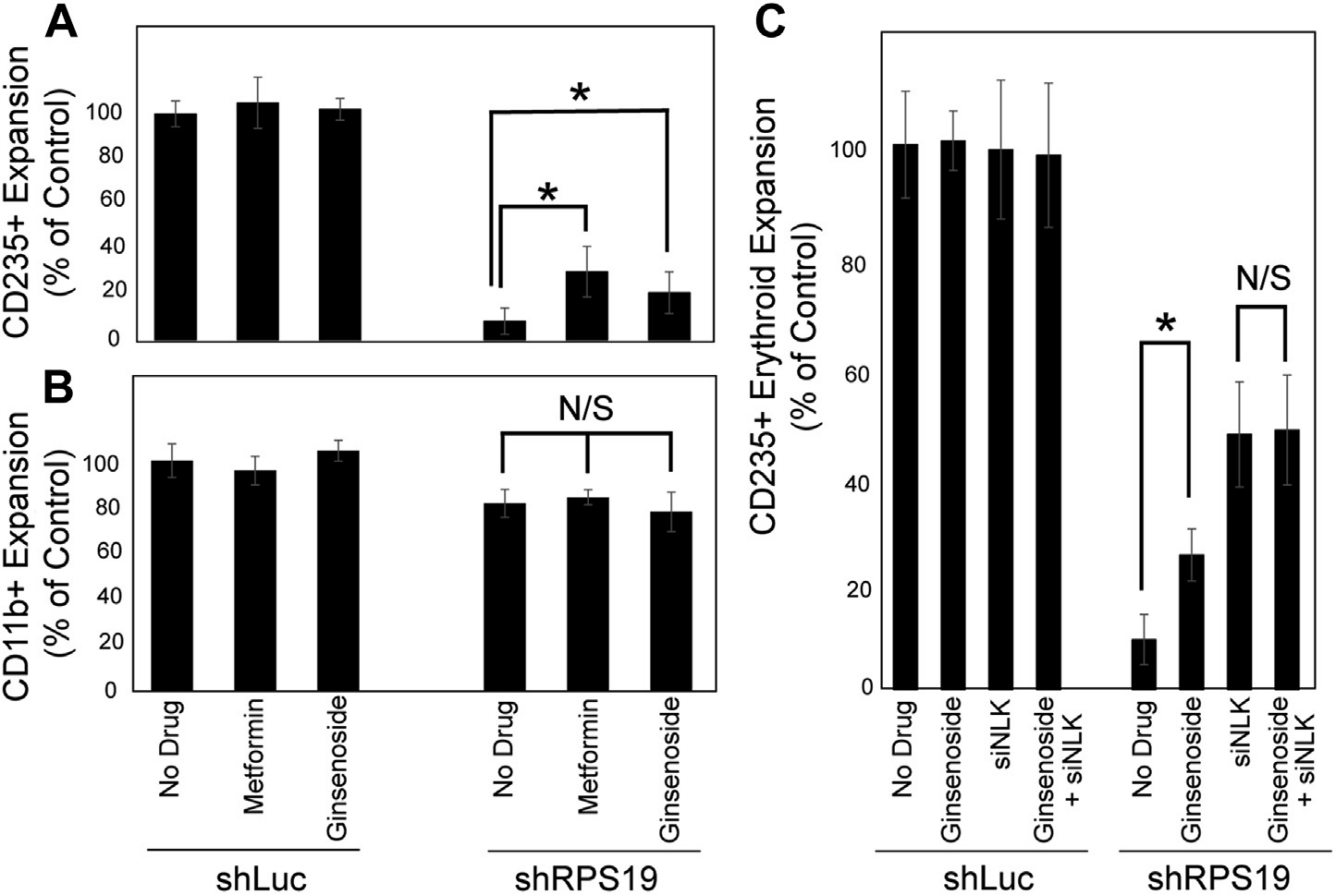
**Ginsenoside Rb1 improves erythroid expansion without impacting myeloid expansion in DBA model.***A*, CD34^+^ HSPCs were transduced with shRNA against control (shLuc) or RPS19 (shRPS19) and after sorting, were differentiated for 14 days in the presence or the absence of 50 mM metformin or ginsenoside Rb1. Cells were counted, and the percentage expressing CD235^+^ erythroid (*A*) and CD11b^+^ myeloid (*B*) was determined by flow cytometry. Obtained values were multiplied to give an overall number that was normalized to the untreated control. Values are presented as a percentage of the untreated control. *C*, CD34^+^ progenitors were transduced with a combination of either shRNA against luciferase or RPS19 (shRPS19) and siRNA against a nontargeting sequence (NT) or NLK (siNLK). After 14 days of differentiation in the presence or the absence of ginsenoside Rb1, cells were counted, and the percentage of cells with surface expression of CD235 and CD11b was determined by flow cytometry to yield the number of CD235^+^ erythroid cells. The total number of cells is expressed as a percentage of the number of each cell type in the control (untreated/shLuc/NT). Data are displayed as means ± SD. Statistics: two-tailed Student's *t* test, significance ∗*p* < 0.05. DBA, Diamond–Blackfan anemia; HSPCs, hematopoietic stem and progenitor cells; NLK, Nemo-like kinase; N/S, not significant.

As the suppression of NLK by miRNAs has been documented (Cichocki *et al.*, 2011 (20); Han *et al.*, 2014 (18); He *et al.*, 2017 (22); Shen *et al.*, 2014 (19); Yan *et al.*, 2016 (21)), we sought to screen a number of common nutritional supplements that have been documented to modulate miRNAs in their ability to improve erythroid expansion in an *in vitro* model of DBA. To induce ribosomal insufficiency, CD34+ HSPCs from human cord blood (CB) were transduced with a shRNA against RPS19 that reduced RPS19 expression by approximately 50% to recapitulate decreased function because of RPS19 mutations in patients with DBA (2, 13, 26, 27). Metformin has been reported to improve erythroid expansion by miRNA-mediated suppression of NLK mRNA expression (13) and was therefore included as a positive control. Similar to metformin (13), 50 mM ginsenoside Rb1 increased erythroid expansion by 2.6-fold (*p* = 0.0488) in RPS19-insufficient hematopoietic progenitor cells but did not significantly impact erythropoiesis in healthy controls (*p* = 0.9129) (Fig. 1*A*). Ginsenoside Rb1 did not impact CD11b^+^ myeloid expansion in control or RPS19 insufficiency (Fig. 1*B*). Efficacy of shRNA against RPS19 was quantified by quantitative RT (qRT)-PCR (Fig. S1*A*). Ginsenoside Rb1 has an EC_50_ value of 2.3 mM with 90% potency achieved at 8.6 mM (Fig. S1*B*). To determine if the ginsenoside Rb1 effect was through the inhibition of NLK, we treated differentiating HSPCs with ginsenoside Rb1 that were transduced with a nontargeting control or siRNA against NLK. Ginsenoside R1 alone or siRNA against NLK alone improved erythroid expansion by 2.7- and 5.1-fold, respectively. The addition of ginsenoside Rb1 when NLK was suppressed by siRNA did not significantly influence erythroid differentiation (5.2-fold, *p* = 0.9037) (Fig. 1*C*), suggesting that the mechanism of action of ginsenoside Rb1 in erythroid rescue in RPS19 insufficiency is through the partial inhibition of NLK.

**Figure 2:**
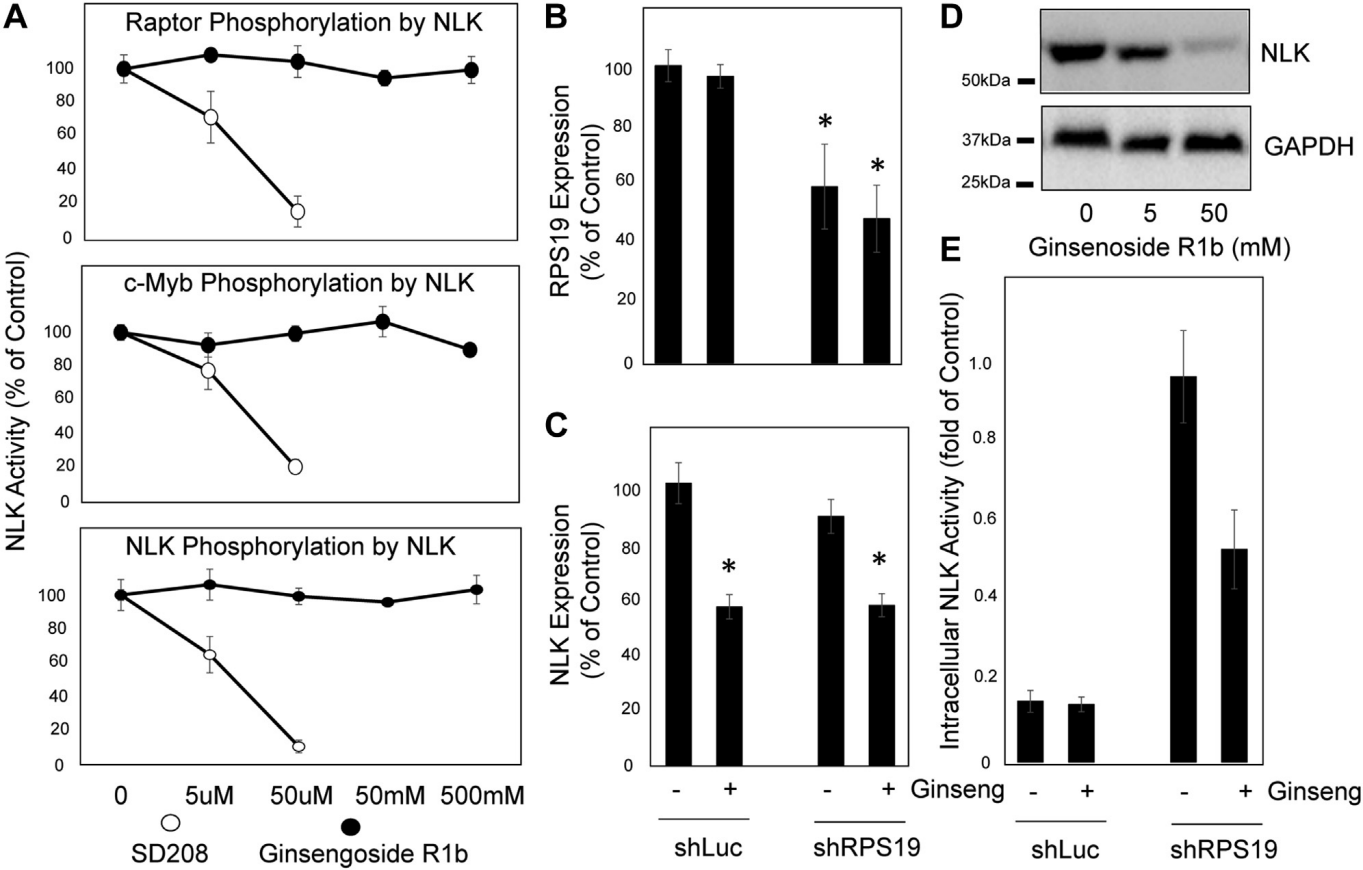
**Ginsenoside Rb1 suppresses NLK expression in erythroid progenitors but does not impact NLK kinase activity.***A*, active NLK was purified from activated Kp53A1 cells and subjected to *in vitro* kinase assay in the presence of 0, 50 nM, 50 μM, 50 or 500 mM ginsenoside Rb1 (*full line* and *closed circles*) or SD208 (*broken line* and *open circles*). The phosphorylation of raptor (*upper*), c-Myb (*middle*), and NLK (*lower*) was determined after 30 min. Control (shLuc) or RPS19-insufficient (shRPS19) progenitors were differentiated in erythroid media alone, vehicle, or vehicle containing 50 mM ginsenoside Rb1 for 5 days. Quantitative RT-PCR was performed to examine RPS19 (*B*) and NLK (*C*) mRNA expression. *D*, NLK (*upper panel*) and GAPDH (*lower panel*) protein expression was assessed by Western blot analysis. *E*, 5000 cells per treatment were lysed, and immunopurified NLK was subjected to *in vitro* kinase assay to determine phosphorylation potential against raptor. Data are displayed as means ± SD. Statistics: two-tailed Student's *t* test, significance ∗*p* < 0.05. NLK, Nemo-like kinase.

To determine if the mechanism of action of ginsenoside Rb1 was through the direct inhibition of NLK kinase activity, we performed *in vitro* kinase assays examining the ability of purified preactivated NLK to phosphorylate three NLK substrates (raptor, c-Myb, and NLK). Even at 500 mM, ginsengoside-6p failed to inhibit NLK kinase activity (Fig. 2*A*—*closed circles*). In contrast, the nonspecific kinase inhibitor SD208 inhibited NLK from phosphorylating NLK, c-Myb, and raptor by 84.3, 79.7, and 89.1%, respectively, at 5 μM (Fig. 2*A*—*open circles*).

In differentiating RPS19-insufficient HSPCs, ginsenoside Rb1 did not influence RPS19 expression. In contrast, 50 mM ginsenoside Rb1 reduced NLK mRNA expression by 43.8% (*p* = 0.0032) and 43.2% (*p* = 0.003) in control and RPS19 insufficiency, respectively (Fig. 2*C*). NLK protein was reduced in differentiating HSPCs by 31.5 and 46.4% at 5 and 50 mM ginsenoside R1b, respectively (Fig. 2*D*). As would be anticipated with reduced NLK expression, NLK immunopurified from equal numbers of ginsenoside Rb1–treated RPS19-insufficient progenitors demonstrated 44.8% reduced NLK kinase activity (*p* = 0.017) (Fig. 2*E*), correlating with the reduced NLK expression (compare Fig. 2, *C* and *E*). As NLK is not activated in control cells, the reduction in expression does not reduce activity, further suggesting that the reduction of NLK activity seen in RPS19 insufficieny is due to a reduction in the number of activated NLK molecules.

**Figure 3:**
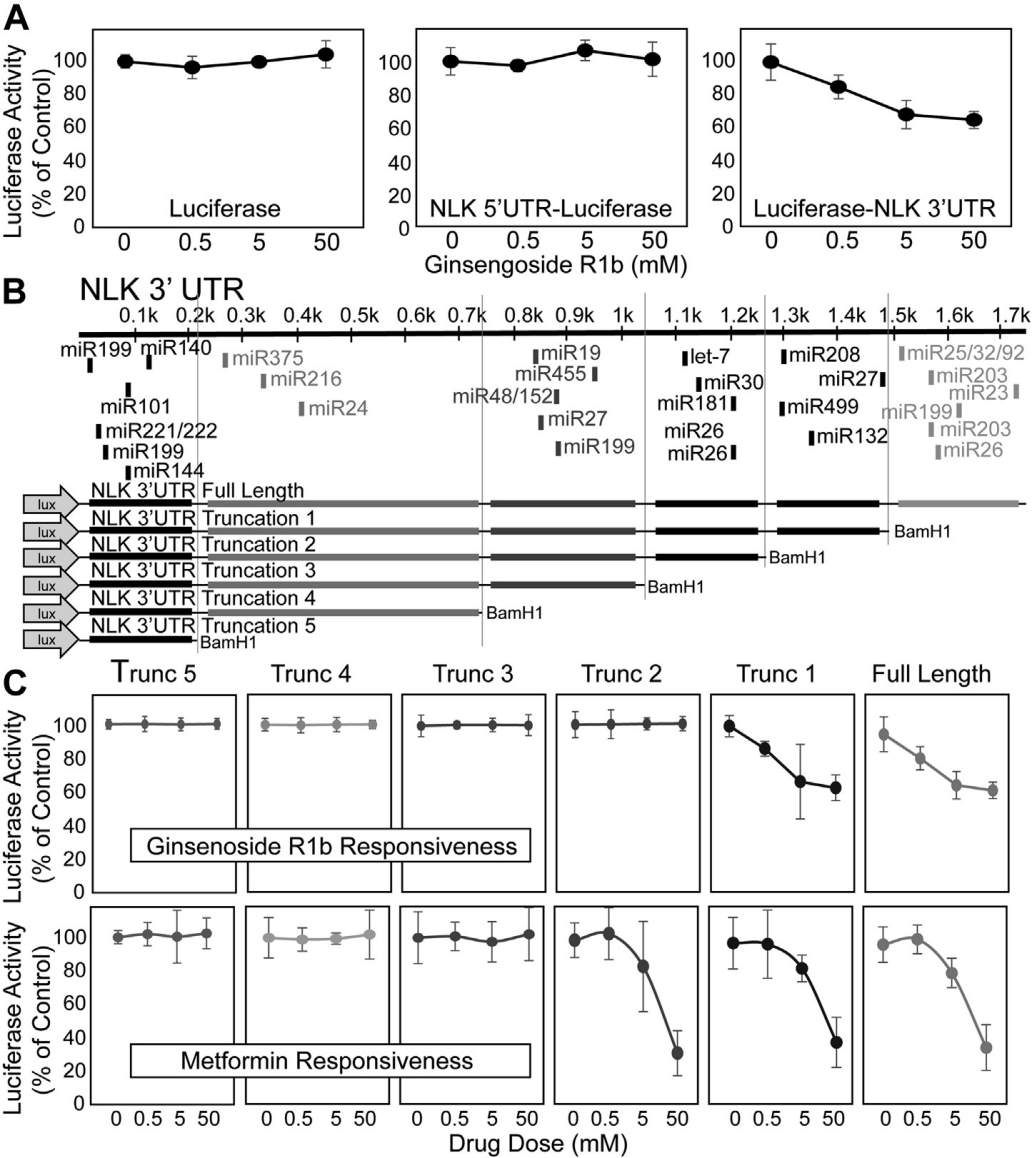
**Ginsenoside Rb1–mediated suppression of NLK expression is mediated through 3′UTR of NLK.***A*, CD34^+^ HSPCs were transduced with luciferase expressed behind a minimal promoter (*left panel*), behind the NLK proximal promoter (*middle panel*), or luciferase immediately upstream of the NLK 3′UTR (*right panel*) and differentiated in the presence of 0, 0.5, 5.0, or 50.0 mM of ginsenoside Rb1 for 72 h. Cells were pelleted and processed for luciferase assay. *B*, schematic representing the many potential miRNA-binding sites within the human NLK 3′UTR (*upper*). Diagrammatic representation of the full-length and five truncated constructs fused to the luciferase gene and engineered to determine the metformin-responsive element within the NLK 3′UTR (*lower*). *C*, after transduction of the indicated 3′UTR truncations, differentiating HSPC cells were treated with indicated concentrations of ginsenoside Rb1 (*upper panels*) or metformin (*lower panels*) for 72 h and assessed for luciferase activity. Data are displayed as means ± SD. Statistics: two-tailed Student's *t* test, significance ∗*p* < 0.05. HSPCs, hematopoietic stem and progenitor cells; NLK, Nemo-like kinase.

Degradation initiated by miRNA typically requires binding of miRNAs to complementary sequences in the mRNA 3′UTR (14). To address whether NLK suppression by ginsenoside Rb1 in RPS19 insufficiency was due to miRNA-mediated events, we fused the NLK 5′UTR or 3′UTR to the luciferase gene and transduced them into ginsenoside Rb1–treated control and RPS19-insufficient differentiating HSPCs. As would be anticipated for a miRNA-mediated event, no downregulation of luciferase activity was observed upon expression of the NLK 5′UTR upstream of the luciferase, but the fusion of the NLK 3′UTR downstream of luciferase leads to a dose-dependent downregulation of luciferase activity (Fig. 3*A*). Luciferase activity was reduced by 35.9% (*p* = 0.0072) at 50 mM ginsenoside Rb1 (Fig. 3*A*) indicating the 3′UTR of NLK is required for NLK mRNA degradation.

The 3′UTR of NLK is 3745 residues and contains at least 30 predicted high potential miRNA-binding sites (TargetScan 7.2; Whitehead Institute for Biomedical Research) (Fig. 3*B*). We generated a series of truncated NLK 3′UTRs fused to the luciferase gene and transduced them into control and RPS19-insufficient differentiating HSPCs. Cells were treated with increasing concentrations of ginsenoside Rb1 or metformin, and luciferase activity was assessed. About 50 mM ginsenoside Rb1 reduced luciferase activity by 35.9% (*p* = 0.0072), whereas metformin reduced luciferase activity by 67.5% (*p* = 0.0184). As previously observed, the ability of metformin to influence the NLK 3′UTR was lost upon truncation of residues between 1007 and 1268 (13). The influence of ginsenoside Rb1 was lost upon truncation of a region between residues 1530 and 1792 (Fig. 3*C*). The predicted miRNA-binding sites contained within this region are miR-499, miR-208, and miR-132.

**Figure 4:**
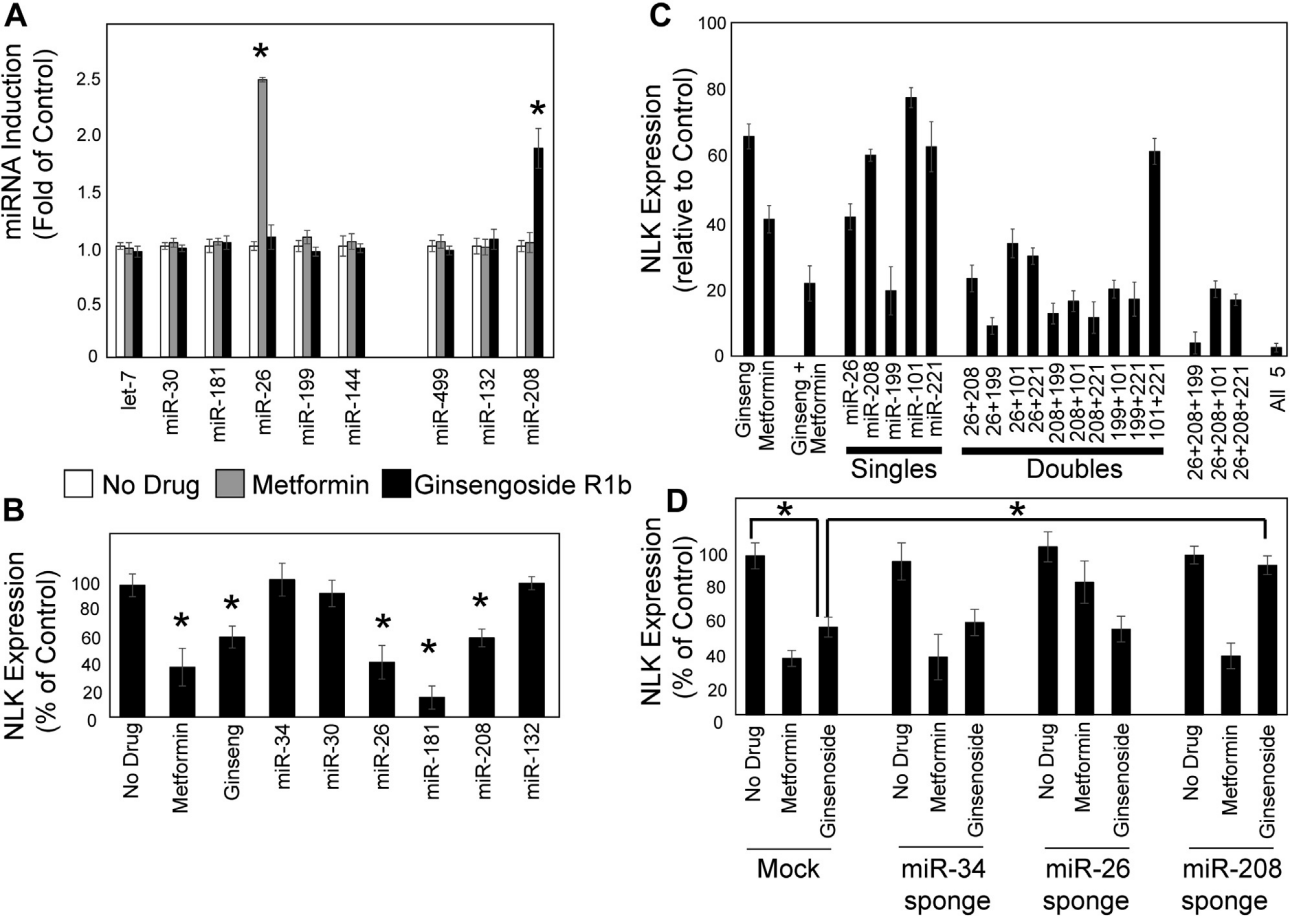
**NLK suppression is through upregulation of miR-208.***A*, human cord blood (CB) CD34^+^ progenitors were differentiated for 4 days in the presence or the absence of 50 mM metformin or ginsenoside Rb1, and the levels of indicated miRNAs were assessed by quantitative RT (qRT)-PCR. *B*, CD34^+^ HSPCs were transduced with the indicated miRNA mimetics or treated with metformin or ginsenoside Rb1 and cultured for 72 h. After lysis, endogenous NLK expression was analyzed by qRT-PCR. Expression of miRNAs was also assessed (Fig. S3*A*). *C*, K562 cells were transfected with indicated combinations of miRNA mimetics or treated with metformin, ginsenoside Rb1, or a combination in the presence of luciferase fused to the NLK 3′UTR. Cells were cultured for 48 h, and luciferase activity was assessed. *D*, CD34^+^ HSPC cells were transduced with either miR-34, miR-26, or miR-208 sponges, prior to being left untreated or treated with metformin or ginsenoside Rb1 for 72 h. Endogenous NLK expression was assessed by qRT-PCR. Expression levels of miRNAs are reported in Fig. S3*B*. Data are displayed as means ± SD. Statistics: two-tailed Student's *t* test, significance ∗*p* < 0.05. HSPCs, hematopoietic stem and progenitor cells; NLK, Nemo-like kinase.

Comparison of miRNA expression in vehicle-, metformin-, and ginsenoside Rb1–treated HSPCs revealed that of the three miRNAs predicted to bind the region of the NLK 3′UTR required for ginsenoside Rb1 sensitivity, only miR-208 was significantly upregulated (*p* = 0.0137) and was induced by 1.9-fold (Fig. 4*A*). None of these miRNAs were induced by metformin since metformin induces miR-26 as published previously (13). The abundances of let-7, miR-30, miR-144, and miR-132 were expressed 40.1-, 2.2-, 5.0-, and 18.3-fold higher than miR-208, whereas miR-208 was expressed 1.6- and 2.1-fold higher than miR-199 and miR-499. MiR-26a (induced by metformin) expression was 1.4-fold higher than miR-208 (Fig. S2).

To confirm miR-208 could downregulate endogenous NLK, we transduced a series of miRNA species into differentiating HSPCs. As has been reported, miR-181 (25), miR-26 (13), miR-199 (18), miR-101 (19), and miR-221 (22) suppressed NLK expression by 85.6% (*p* = 0.0035), 58.7% (*p* = 0.0155), 80.8% (*p* = 0.0026), 23.0% (*p* = 0.0056), and 37.7% (*p* = 0.0129), respectively. Expression of miR-208 reduced NLK expression by 40.2% (*p* = 0.0093), which correlates closely with the influence of ginsenoside Rb1 (34.6%) (Fig. 4*B*). Expression of miRNAs is quantified by qRT-PCR (Fig. S3*A*).

We further examined if the expression of various combinations of these miRNAs suppressed NLK expression more than single miRNAs alone. MiR-199 suppressed NLK expression better than other miRNA species, reducing NLK expression to 19.2% of control. Expression of two or more miRNAs generally decreased NLK expression, although the synergistic effect varied with each combination. Maximal suppression by a single miRNA was 80.8% (by miR-199) with a maximal effect observed upon combining all five miRNAs with NLK expression reduced to 2.2% (*p* = 0.0003) of control (Fig. 4*C*). The combined properties did not strongly correlate with the suppressive effect of each individual miRNA, suggesting a complex interplay between miRNAs, the degradative machinery and the NLK 3′UTR. Combined ginsenoside Rb1 and metformin treatment further decreased NLK expression down to 21.4% of control, from 65.4% and 40.6% with ginsenoside Rb1 or metformin alone (*p* = 0.0048 and 0.025, respectively).

Having established that miR-208 is upregulated in response to ginsenoside Rb1 (Fig. 4*A*) and that miR-208 suppresses NLK expression (Fig. 4*B*), we sought to test the hypothesis that ginsenoside Rb1–mediated upregulation of miR-208 is responsible for ginsenoside Rb1–mediated suppression of NLK expression. To address this, various inhibitors of miRNAs (miRNA sponges) were transduced into ginsenoside Rb1– or metformin-treated differentiating HSPCs. Expression of miRNAs was assessed by qRT-PCR (Fig. S3*B*). As anticipated, miR-26 inhibition prevented metformin-induced NLK suppression (Fig. 4*D*). Ginsenoside Rb1 treatment reduced endogenous NLK expression by 44.8% (*p* = 0.0065), but inhibition of miR-208 rescued this back to 94.1% of control (*p* = 0.0076) (Fig. 4*D*). This indicates that ginsenoside Rb1 indeed suppresses NLK expression through the induction of miR-208.

**Figure 5:**
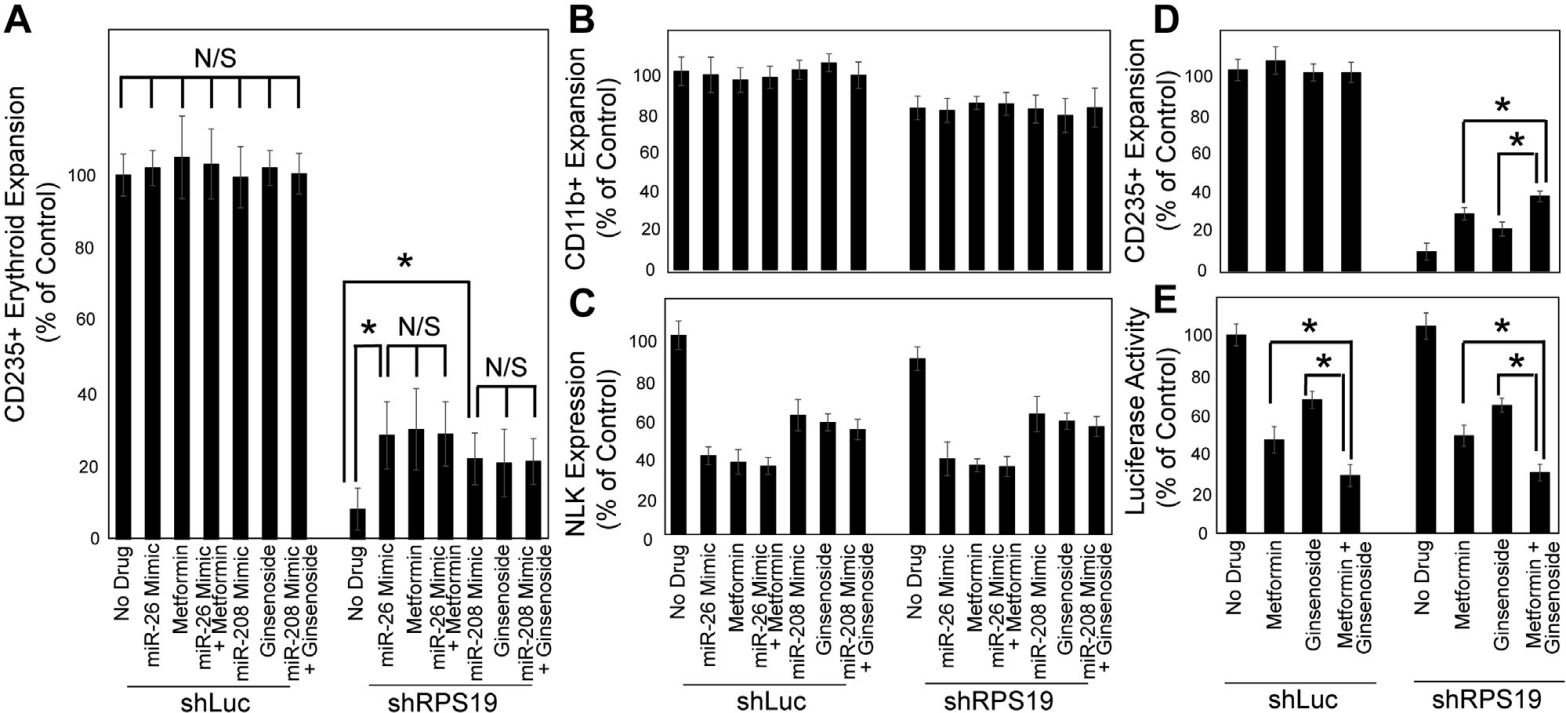
**Erythroid improvement by ginsenoside Rb1 in DBA model is through miR-208.***A*, human CD34^+^ cells were transduced with shRNA against a control (shLuc) or RPS19 (shRPS19) and transfected with a miR-26 or miR-208 mimetics or a mock control. After differentiation in the absence or the presence of metformin or ginsenoside Rb1 for 12 days, cells were counted and the percentage of CD235^+^ erythroblasts determined by flow cytometry. The calculated CD235^+^ population of each treatment group is represented as a percentage of the untreated control group (*upper*). *B*, the population of CD11b^+^ myeloid cells was similarly assessed. *C*, in parallel, quantitative RT-PCR was performed to determine mRNA expression of NLK. RPS19, miR-26, and miR-208 expression is shown in Fig. S4. *D*, CD34+ HSPCs were transduced with nontargeting or shRNA against RPS19 and luciferase fused to the NLK 3′UTR. Cells were treated with 50 mM metformin and/or ginsenoside Rb1 for 12 days, and CD235+ erythrocytes were assessed by flow cytometry. *E*, cell pellets were also assessed for luciferase activity as a surrogate for NLK expression. Data are displayed as means ± SD. Statistics: two-tailed Student's *t* test, significance ∗*p* < 0.05. DBA, Diamond–Blackfan anemia; HSPCs, hematopoietic stem and progenitor cells; NLK, Nemo-like kinase; N/S, not significant.

Having confirmed ginsenoside Rb1–mediated upregulation of miR-208 is responsible for the suppression of NLK in differentiating HSPCs, our final goal was to assess if upregulation of miR-208 indeed accounted for the improved erythropoiesis in response to ginsenoside Rb1. CB CD34^+^ HSPCs were transduced with control or shRNA against RPS19 in combination with metformin or ginsenoside Rb1 and/or miR-26 or miR-208. As anticipated, miR-26 restored erythropoiesis in RPS19 insufficiency to a similar extent as metformin. Similarly, miR-208 increased erythroid expansion by 2.65-fold (*p* = 0.0473) and ginsenoside Rb1 increased it by 2.52-fold (*p* = 0.1416). As combining miR-208 with ginsenoside Rb1 did not significantly increase erythropoiesis compared with either treatment alone (2.57-fold to 2.65-fold and 2.52-fold, respectively) (*p* = 0.8801 and 0.9432) (Fig. 5*A*), it is highly probable that the ginsenoside Rb1 rescue is mediated primarily through miR-208 upregulation. No significant impact on CD11b^+^ myeloid expansion was observed in response to drug or miRNA manipulation (Fig. 5*B*).

While erythroid expansion is improved in RPS19 insufficiency, there is no impact on healthy controls (Fig. 5*A*), but examination indicates that NLK expression is similarly suppressed by each treatment in both healthy controls and RPS19 insufficiency (Fig. 5*C*). This reflects the fact that NLK is dispensable for healthy erythropoiesis and only impacts erythroid expansion once it is activated in ribosomal insufficiency (2, 13). RPS19 and miR-208 and miR-26 expression were assessed (Fig. S4). As the combination of ginsenoside Rb1 and metformin further suppressed NLK expression from 34.6% and 59.4% alone to 78.6% (Fig. 4*C*), we sought to determine if combining metformin and ginsenoside Rb1 could act synergistically to improve erythroid expansion in RPS19 insufficiency. Ginsenoside Rb1 (50 mM) and metformin (50 mM) were combined and added to HSPCs transduced with shRNA against RPS19 or control. The combined treatment further enhanced erythroid expansion in RPS19 insufficiency from 20.9% and 28.4% when cells were treated with either ginsenoside Rb1 or metformin alone, to 37.1% (*p* = 0.0091 and 0.0306) when treated in combination (Fig. 5*D*). No significant toxicity was observed in healthy erythroid (Fig. 5*D*). NLK 3′UTR-regulated luciferase activity was inversely proportionally reduced with cotreatment significantly decreasing NLK expression compared with either drug alone (*p* = 0.005 and 0.0164, respectively) (Fig. 5*E*). While this study is focused on documenting the mechanism of ginsenoside Rb1–mediated erythroid rescue in DBA models, these data suggest that combining ginsenoside Rb1 (or other modulators of miR-208 expression) may have additional therapeutic benefit to other therapies that may suppress NLK expression/activity by other mechanisms. While combination therapy was not additive, significant synergy was observed with combination index values of 0.83, 0.69, and 0.58 at 50%, 70%, and 90% of maximal erythroid rescue values (Fig. S5).

Collectively, our data indicate that ginsenoside Rb1 induces miR-208 upregulation in differentiating erythroid progenitors that suppresses NLK expression. In RPS19 insufficiency, the reduced NLK expression protects progenitors from the impacts of NLK activation and improves erythroid expansion.

## Discussion

The suppression of NLK by ginsenoside Rb1 significantly improves erythroid expansion in models of DBA, but rescue is not complete. Indeed, even suppression of NLK expression by siRNA or shRNA, or inhibition of NLK activity by broad-acting kinase inhibitors, does not restore erythropoiesis to the same level as healthy controls (2). However, clinical studies with steroid treatment have shown that even raising red blood cell counts incrementally can significantly improve patient outcomes, such as transfusion independence or at least reducing transfusion regularity (11, 12, 28). Evaluating the exact clinical benefit to patients with DBA will require *in vivo* and clinical studies and will likely be dependent on a myriad of factors, including the specific gene mutation and severity of the disease.

It is interesting that both ginsenoside Rb1 and miR-208 reduce NLK expression less than metformin or miR-26a. While it is difficult to make assumptions about the confirmation of miRNAs binding mRNA, it is possible that miR-208 is less efficient at binding the consensus site or that bound miR-208 is less efficient at recruiting the degradative machinery. MiR-26a expression is more abundant than miR-208 in control HSPCs (Fig. S2). Coupled to the less robust induction of miR-208 in response to ginsenoside Rb1 (Fig. 4*A*), it is plausible that the reduced abundance of miR-208 contributes to NLK suppression being more sensitive to metformin. As miR-181 binding appears to be evolutionarily conserved to dramatically downregulate NLK (sequence preserved across human, mouse, and zebrafish), the fact that the miR-26a binding site is in close proximity may contribute to a more dramatic downregulation in response to the miR26a-inducing metformin.

A positive and negative aspect of miRNAs is the tissue specificity of their induction (14, 16, 23). In this case, that may mean systemic treatment with ginsenoside Rb1 will suppress NLK expression in HSPCs but not in other tissues. In contrast, these compounds may induce other miRNAs in other tissues with deleterious impacts. TargetScan 2.0 indicates that miR-208 potentially targets 211 mRNAs but whether these mRNAs are expressed in tissues ginsenoside Rb1 induces miR-208, or the extent of influence miR-208 has on these mRNAs, has not been evaluated. However, as millions of people use ginseng as a nutritional supplement without adverse events (29), this seems unlikely. A further consideration is generating a high enough concentration of ginsenoside Rb1 at the sites of hematopoiesis in the bone marrow. With an EC_50_ of 2.3 mM, the concentration of ginsenoside required for efficient erythroid rescue is high and may constitute problems for dosing in patients. Ginseng is well tolerated, and doses as high as 4.5 g/day have been given orally with no reported toxicity (29). Unfortunately, orally ingested ginsenosides are not efficiently absorbed into blood plasma and are rapidly cleared (30). Intravenous and intramuscular administration provides bioavailable ginsenosides, and single doses of at least 75 mg are well tolerated (31). In mice, sustained daily intraperitoneal injections of 0.2 ml of 4 mg/ml ginsenoside Rb1 demonstrated no adverse effects and enhanced hematopoietic function and dendritic cell differentiation (32), suggesting it is possible to introduce high enough concentrations of ginsenoside Rb1 in the bone marrow to influence HSPC differentiation. Antibody-targeted nanoparticles delivering ginsenoside Rb1 directly to the HSPCs may overcome systemic and local concentration issues.

Alternatively, aptamer-fused miR-208, or a combination of miRNAs, could be delivered specifically to HSPCs. Indeed, this application has already been validated with aptamers targeted to c-kit bound to miR-26a and delivered to HSPCs to attenuate toxicity of 5′fluorouracil and carboplatin in a mouse model (33). Indeed, our data suggest that delivering a cocktail of miRNAs specifically to HSPCs may have significant therapeutic benefit. As the ability to predict the efficacy of various combinations of miRNAs did not correlate closely to the efficacy of miRNAs administered individually, determining optimal doses and combinations may require extensive experimentation. Likewise, evaluating potentially deleterious off-target effects of ginsenoside Rb1, miR-208, or combination therapies requires further study.

The application of miRNA-mediated suppression of NLK is not limited to DBA. Poor prognosis of a number of carcinomas, osteosarcomas, and neuroblastomas has all been attributed to elevated NLK expression (3, 4, 5, 6, 7, 8, 9, 10). The ability to suppress NLK may be one reason ginseng is thought to have benefits for colorectal and lung cancers (34, 35, 36, 37). While the benefits of developing small-molecule kinase inhibitors is well recognized in modern medicine, in the event of highly conserved kinases, targeting unique attributes of the target may offer higher specificity and less off-target effects. We propose that induction of miRNAs that target NLK for degradation is one such approach.

## Experimental procedures

### Cell culture

As described previously (2, 13, 26), primary human CD34^+^ HSPCs were purified from CB (New York Blood Center) by using magnetic-activated cell sorting (Miltenyi Biotec) and were cryopreserved. Upon thawing, cells were cultured in x-Vivo15 media (Lonza) containing 10% fetal bovine serum, fms-related tyrosine kinase 3 (50 ng/ml), thyroid peroxidase (50 ng/ml), interleukin-3 (20 ng/ml), interleukin-6 (20 ng/ml), and stem cell factor (50 ng/ml) and differentiated for 12 days. Kp53A1 cells were obtained from Javier Leon and cultured in Dulbecco's modified Eagle's medium supplemented with 10% fetal bovine serum at 37 or 32 °C. shRNA against RPS19 (or luciferase) with approximately 50% knockdown efficiency was cloned into shRNA-carrying vectors (pLVTH) coexpressing GFP. Luciferases, or luciferases fused to various permutations of the NLK 3′ or 5′UTRs, were cloned in pcDNA3.1 expressing red fluorescent protein (RFP).

### Lentiviral transduction

CD34^+^ cells were transduced as published (26) with lentivirus expressing shRNA against RPS19, or luciferase (Luc). Cotransduction with lentivirus-expressing luciferase fused to various truncations of NLK 5′UTR or 3′UTR or miRNA mimics or sponges was also performed. Virus coexpressed GFP, RFP, mCherry, or puromycin to enable selection.

### Ginsenoside Rb1, metformin, and miRNAs

Ginsenoside Rb1 and metformin were purchased from SelleckChem and added to cells at indicated concentrations with a final dimethyl sulfoxide concentration of 0.5%. MISSION synthetic miRNA inhibitors and mimetics were purchased from Sigma–Aldrich, and mirVana miRNA inhibitors were purchased from Thermo Fisher Scientific. All were transduced according to manufacturer's instructions. EC_50_ values were calculated by Quest Graph EC50 Calculator, AAT Bioquest, Inc.

### Flow cytometry

Cells were incubated with human Fc receptor–binding inhibitor (#14-9161-73; eBioscience) followed by primary antibodies CD235-APC (#306607; BioLegend) and CD11b-PE/Cy5 (#101209; BioLegend). Data were collected on a DxP10 flow cytometer (Cytek) and analyzed by using FlowJo Software, version 9.7.2 (FlowJo, LLC).

### Kinase assays, Western blotting

NLK kinase activity was performed as published (2). For Western blotting, antibodies against NLK (#AB97642; Abcam; 1:1000 dilution) and GAPDH (#MAB374; Millipore; 1:10,000) were used according to manufacturer's instructions.

### qRT-PCR

mRNA was quantified as described (2). miRNA was quantified using TaqMan Small RNA Assays (Applied Biosystems) as per manufacturer's directions and normalized to snoRNA234.

### Luciferase assay

The NLK minimal promoter (1019 5′ nucleotides) and or NLK 3′UTR (1885 3′ nucleotides) was cloned upstream or downstream, respectively, of firefly luciferase and transfected into K562 cells. Transfection efficiency was normalized by RFP expression, and firefly luciferase activity was determined by Luciferase Assay Reagent II from Dual-Luciferase Reporter Assay System (Promega). Luminescence was assessed using a Synergy H1 hybrid multimode microplate reader (BioTek). Mutations and truncations in NLK 3′UTR were introduced using QuikChange II XL Site-directed Mutagenesis (Agilent).

### Statistics

*p* Values for statistical significance were obtained by using a paired Student's *t* test. Significance was designated as *p* < 0.05. The data are representative of at least three independent experiments. When possible, variability between replicates was normalized for by designating values of controls to 100% (or onefold), and comparing variables against that. Combinatorial index (drug combinations) was determined using CompuSyn software (ComboSyn, Inc).

## Data availability

Further information and requests for resources and reagents should be directed to and will be fulfilled by the lead contact, Kathleen Sakamoto (kmsakamo@stanford.edu). Reagents generated in this study will be made available on request, but we may require a payment and/or a completed Materials Transfer Agreement if there is potential for commercial application. This study did not generate/analyze datasets or code.

## Supporting information

This article contains supporting information.

## Conflict of interest

The authors declare that they have no conflicts of interest with the contents of this article.
