# Supplementary material for: The active component of ginseng, ginsenoside Rb1, improves erythropoiesis in models of Diamond–Blackfan anemia by targeting Nemo-like kinase
Source: J Biol Chem. 2021 Jul 21;297(3):100988. doi: 10.1016/j.jbc.2021.100988 (PMC8379498; doi:10.1016/j.jbc.2021.100988)

Supplemental Figures

Figure S1. Effect of ginsenoside on erythroid expansion in RPS19-insufficiency.

(A) CD34$^+$ HSPCs were transduced with shRNA against control (shLuc) or RPS19 (shRPS19) and after sorting, were differentiated for 14 days in the presence or absence of 50mM metformin or ginsenoside Rb1. RPS19 mRNA expression was quantified by qRT-PCR. (B) CB CD34+ HSPCs were treated as above with concentrations of ginsenoside Rb1 ranging from 1 nM to 1M. Cells were counted and the percentage expressing CD235 was determined by flow cytometry. Obtained values were multiplied to give an overall number that was normalized to the untreated control. An EC50 curve was generated and the EC50 value was calculated. Corresponds with Figure 1.

Figure S2. Expression of miRNA species in CD34+ HSPCs.

CD34+ HSPCs were subjected to qRT-PCR to assess expression of let-7, miR-30, miR-144, miR-132, miR-199, miR-499 and miR-26a. MiRNAs were normalized to snoRNA234 prior to having their expression values expressed relative to miR-208. Corresponds with Figure 4A.

Figure S3. Impact of miRNAs on NLK expression in RPS19-insufficiency.

(A) CD34$^+$ HSPCs were transduced with the indicated miRNA mimetics, or treated with metformin or ginsenoside Rb1, and cultured for 72 hrs. After lysis, expression of the indicated miRNAs was analyzed by qRT-PCR. Corresponds with Figure 4B. (B) CD34$^+$

HSPCs cells were transduced with either miR-34, miR-26 or miR-208 sponges, prior to being left untreated or treated with metformin or ginsenoside Rb1 for 72 hrs. Indicated miRNA expression was assessed by qRT-PCR. Corresponds with Figure 4C.

Figure S4. Impact of miRNAs on NLK expression in RPS19-insufficiency.

Human CD34$^+$ cells were transduced with shRNA against a control (shLuc) or RPS19 (shRPS19) and transfected with a miR-26 or miR-208 mimetics or a mock control. After differentiation in the absence or presence of metformin or ginsenoside Rb1 for 12 days, the expression of miR-208, miR-26 and RPS19 was determined by qRT-PCR. Corresponds with Figure 5.

Figure S5. Ginsenoside Rb1 and metformin synergistically improve erythropoiesis in RPS19-insufficiency.

Ginsenoside Rb1 and metformin concentrations are presented on the X and Y axis respectively and EC50, EC70 and EC90 values for each compound are plotted (top). Indicated doses of metformin were incubated in the presence of EC50 (triangle), EC70 (square) or EC90 (circle) ginsenoside Rb1. Effect is plotted as the extent of erythroid expansion relative to ginsenoside-rescued erythropoiesis (bottom).

Supplementary Figure 1

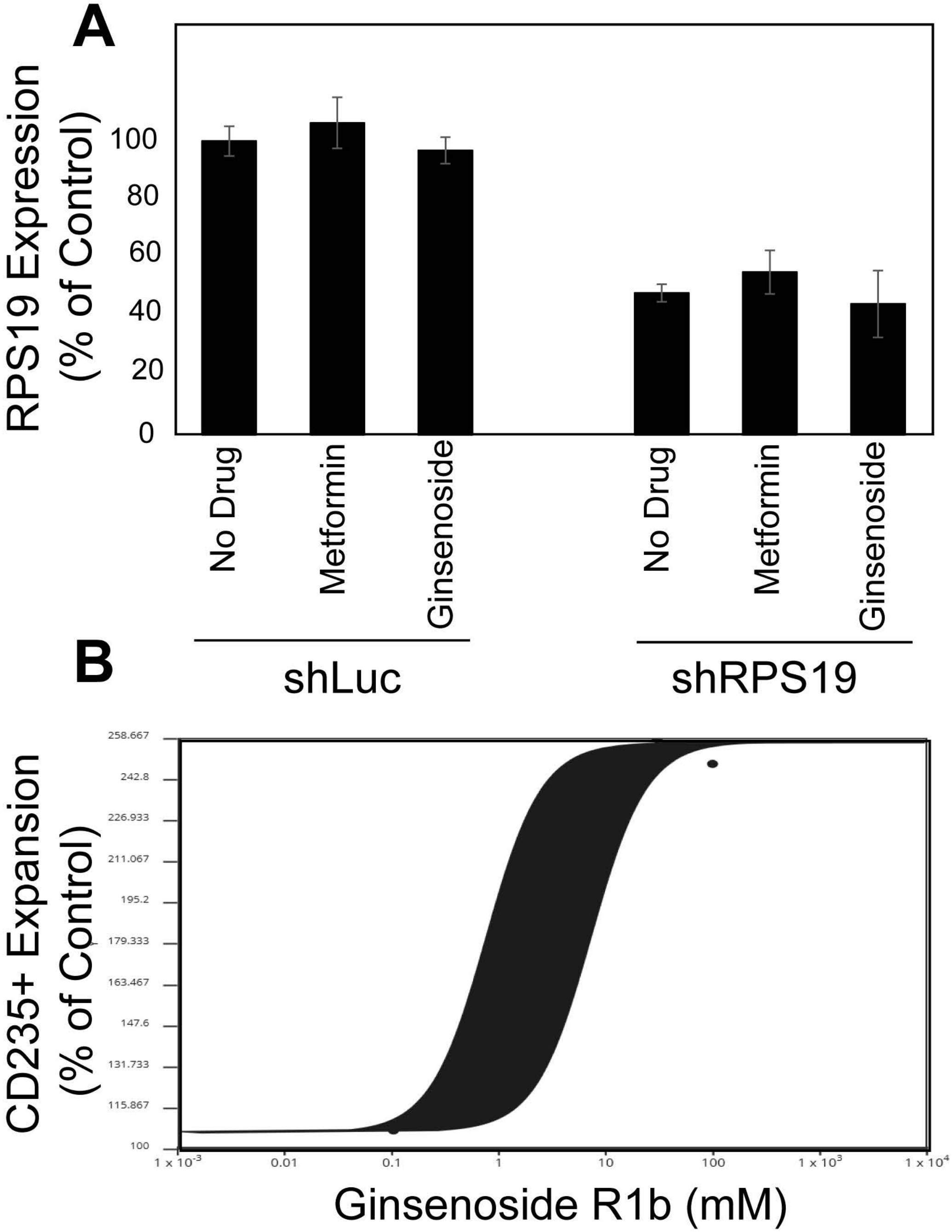

Supplementary Figure 2

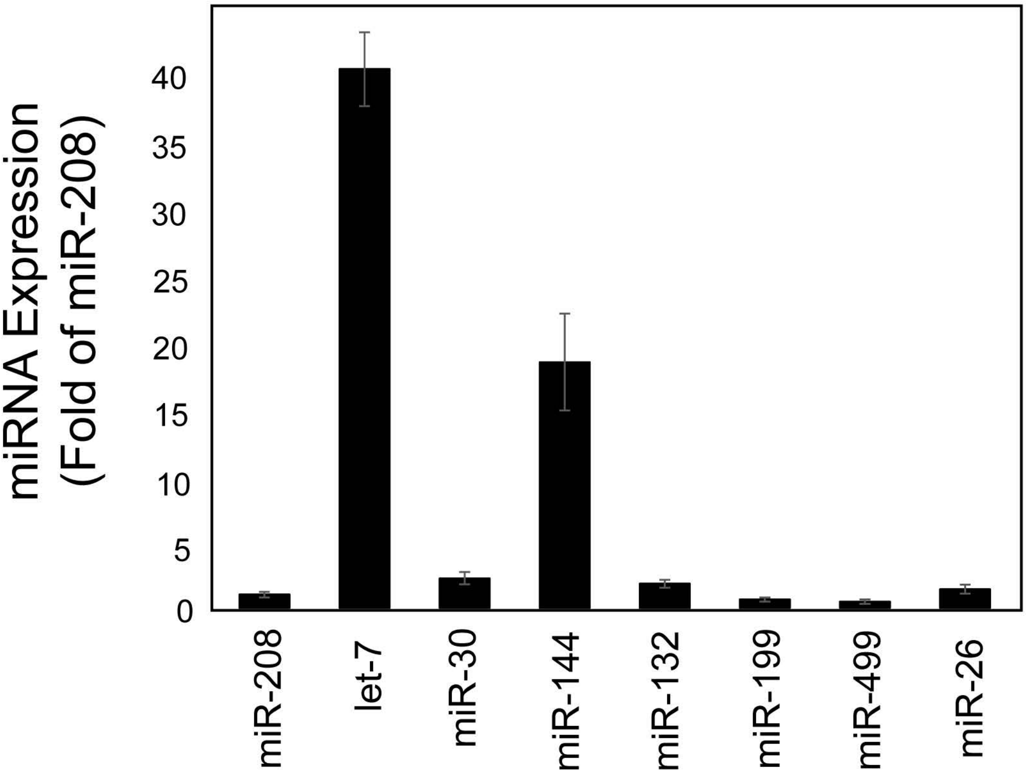

Supplementary Figure 3

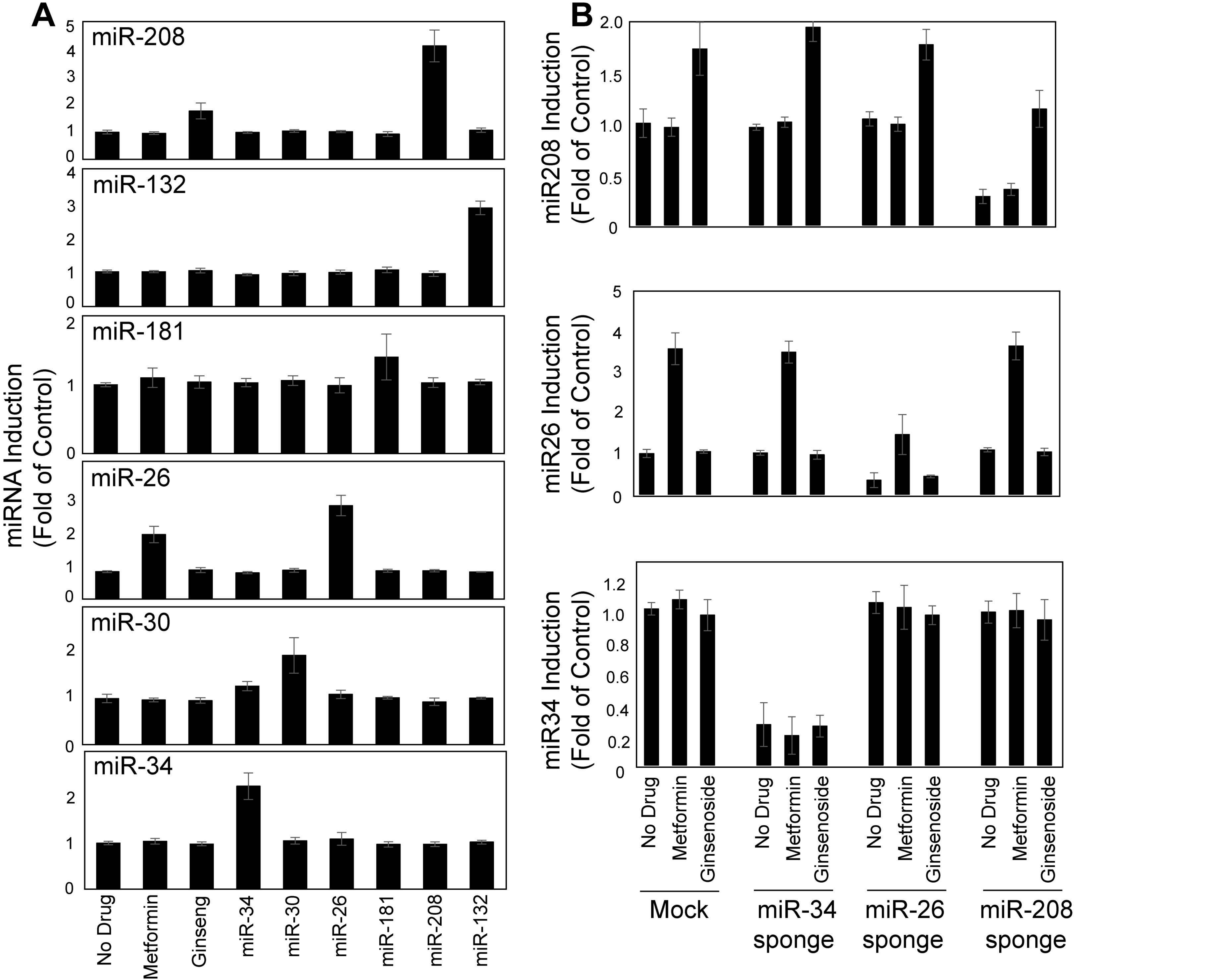

Supplementary Figure 4

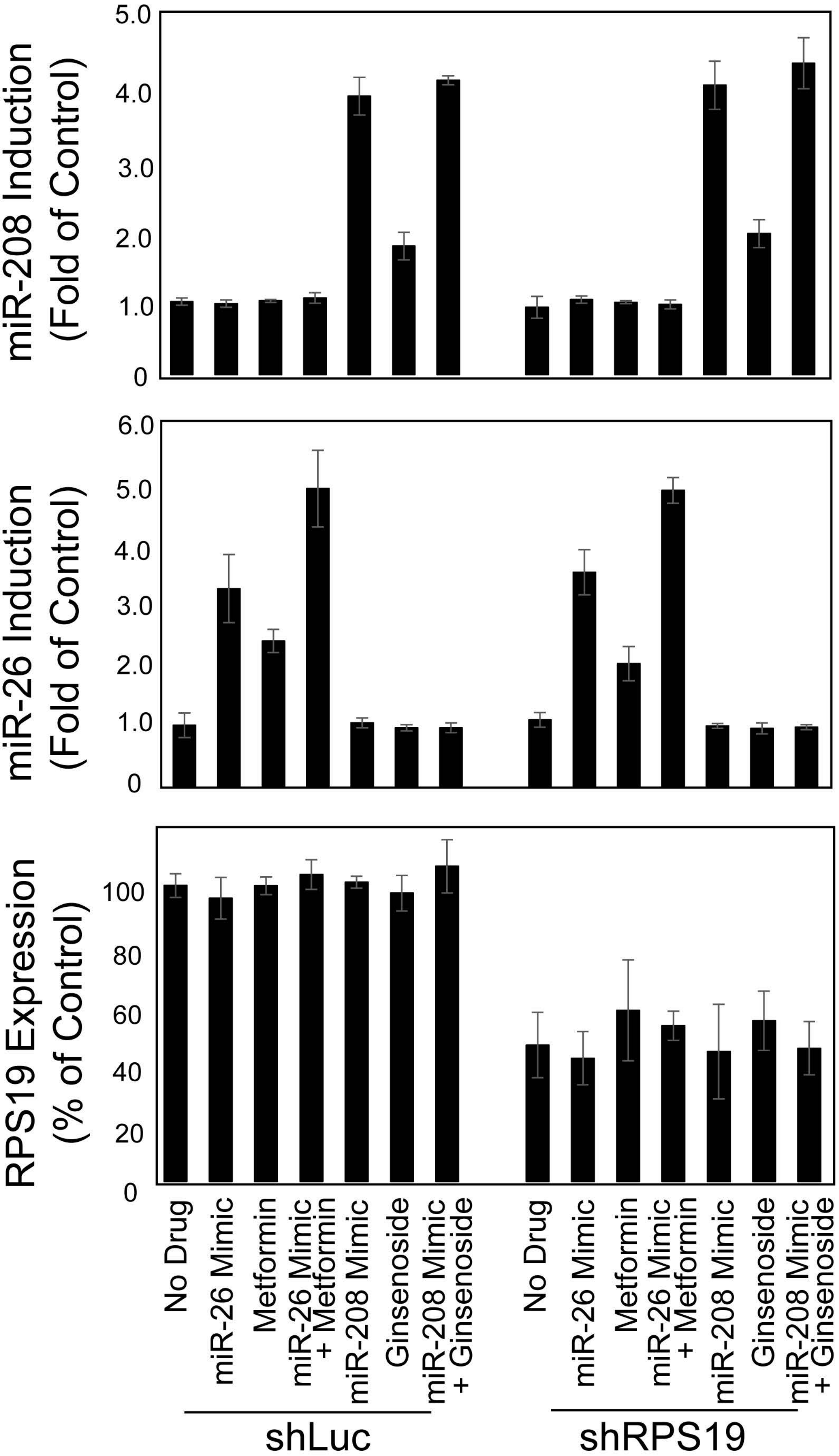

Supplementary Figure 5

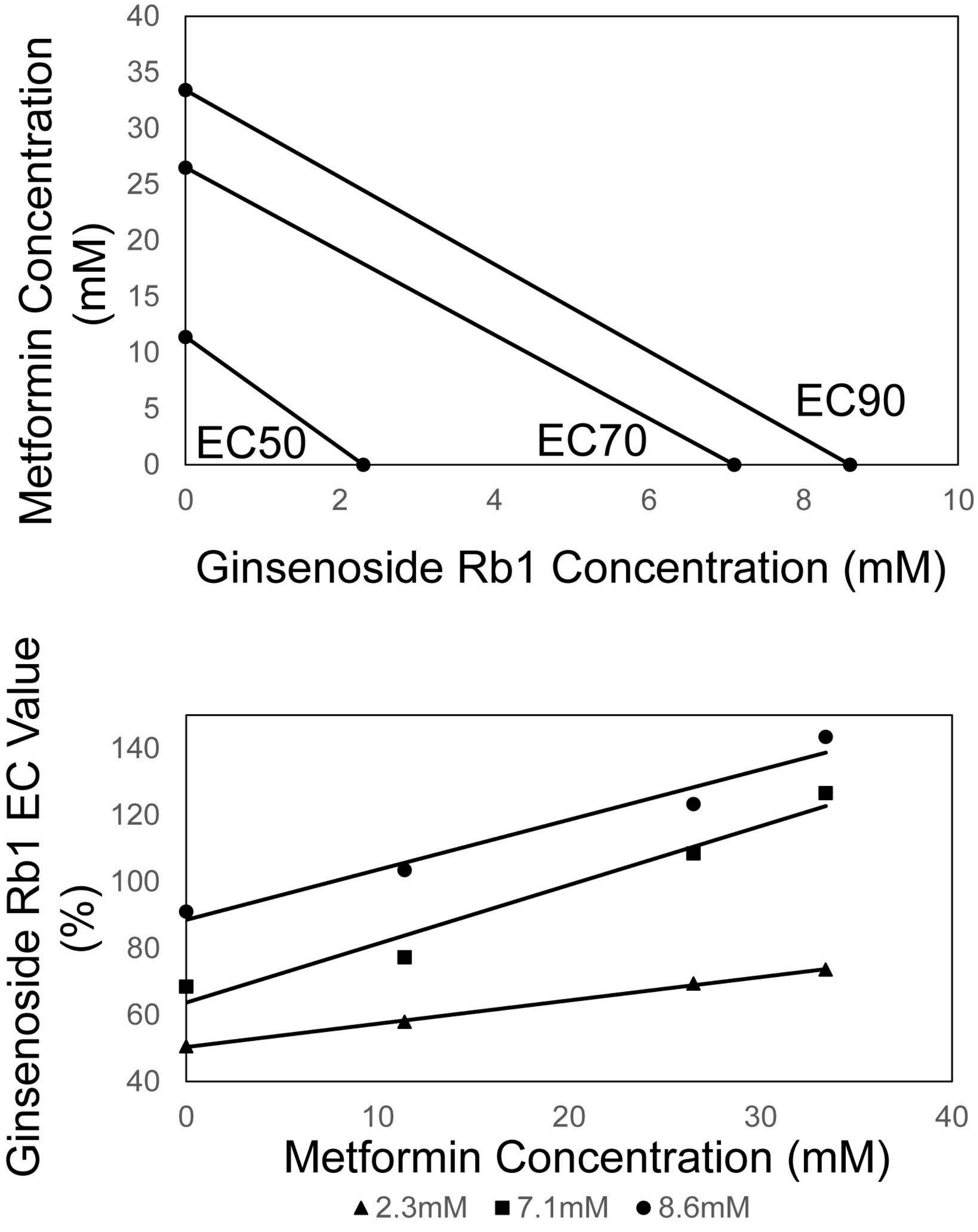

Supplement: Supplemental Figures S1–S5 [file mmc1.pdf]
